# Small Interfering RNAs as Critical Regulators of Plant Life Process: New Perspectives on Regulating the Transcriptomic Machinery

**DOI:** 10.3390/ijms26041624

**Published:** 2025-02-14

**Authors:** Marta Puchta-Jasińska, Paulina Bolc, Aleksandra Pietrusińska-Radzio, Adrian Motor, Maja Boczkowska

**Affiliations:** Plant Breeding and Acclimatization Institute—National Research Institute, 05-870 Radzików, Poland; a.pietrusinska@ihar.edu.pl (A.P.-R.); a.motor@ihar.edu.pl (A.M.); m.boczkowska@ihar.edu.pl (M.B.)

**Keywords:** small interfering RNA, gene silencing, abiotic and biotic stress

## Abstract

Small interfering RNAs (siRNAs) are a distinct class of regulatory RNAs in plants and animals. Gene silencing by small interfering RNAs is one of the fundamental mechanisms for regulating gene expression. siRNAs are critical regulators during developmental processes. siRNAs have similar structures and functions to small RNAs but are derived from double-stranded RNA and may be involved in directing DNA methylation of target sequences. siRNAs are a less well-studied class than the miRNA group, and researchers continue to identify new classes of siRNAs that appear at specific developmental stages and in particular tissues, revealing a more complex mode of siRNA action than previously thought. This review characterizes the siRNA classes and their biogenesis process and focuses on presenting their known functions in the regulation of plant development and responses to biotic and abiotic stresses. The review also highlights the exciting potential for future research in this field, proposing methods for detecting plant siRNAs and a bioinformatic pathway for identifying siRNAs and their functions.

## 1. Introduction

Next-generation sequencing has facilitated the discovery of novel regulators of plant development, namely non-coding RNAs. Non-coding RNA classes, which have many vital functions in the cell, have received particular attention. In animals and plants, the protein-coding regions comprise only a tiny fraction of the genome, and approximately 90% comprises non-coding regions [1,2,3]. Non-coding RNAs (ncRNAs) are molecules derived from different areas in plant genomes and are a diverse family of RNA molecules. Despite being transcribed by RNA polymerase, these molecules lack codons for protein synthesis, differentiating them from regular RNAs [4]. One of the classification methods for non-coding RNAs (ncRNAs) is based on their functional characteristics. They can be subdivided into two primary categories: structural/housekeeping ncRNAs and regulatory ncRNAs. Structural ncRNAs such as ribosomal RNAs (rRNAs), transfer RNAs (tRNAs), small nuclear RNAs (snRNAs), and small nucleolar RNAs (snoRNAs) serve essential structural functions within the cell. In contrast, regulatory ncRNAs, including small RNAs (sRNAs), intermediate-size ncRNAs (im-ncRNAs), long noncoding RNAs (lncRNAs), and circular RNAs (circRNAs), are crucial for gene regulation. Among these regulatory ncRNAs, small RNAs form a subclass that is further divided into microRNAs (miRNAs) and small interfering RNAs (siRNAs) [5]. Most non-coding RNAs have regulatory functions in response to changing environmental conditions or signals that trigger developmental processes. They are usually tissue- and temporal-specific molecules.

**Figure 1:**
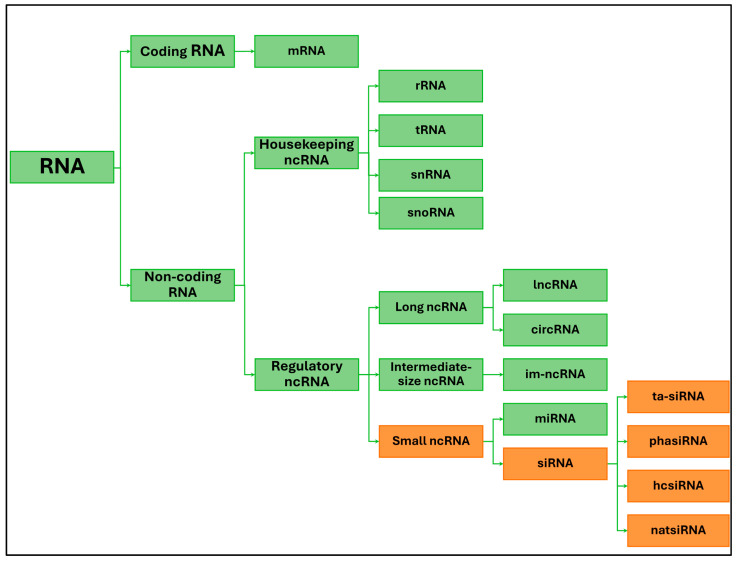
The plant RNA universe: messenger RNA (mRNA), non-coding RNA (ncRNA), ribosomal RNA (rRNA), transfer RNA (tRNA), small nuclear RNA (snRNA), and small nucleolar RNA (snoRNA), long noncoding RNA (lncRNA), intermediate-size ncRNA (im-ncRNA), micro RNA (miRNA), small interfering RNA (siRNA), natural antisense siRNA (natsiRNA), trans-acting siRNA (ta-siRNA), heterochromatic siRNA (hcsiRNA), phase-acting siRNA (phasiRNA).

Small interfering RNAs (siRNAs) are 21, 22, and 24 nt molecules. They are classified into the classes of trans-acting siRNAs (tasiRNAs), phase-acting siRNAs (phasiRNAs), heterochromatic siRNAs (hcsiRNAs), and natural antisense siRNAs (nat-siRNAs) (Figure 1) [6]. siRNAs are a subclass of small regulatory RNAs formed from RNA duplexes. Duplexes can be formed during viral RNA replication by the transcription of inverted repeat sequences or by the action of endogenous RNA-dependent RNA polymerases [7].

Like miRNAs, siRNAs bind to proteins, thereby facilitating the degradation of complementary mRNA molecules. This results in the cessation of translation [8,9] (Table 1). The distinction between these non-coding RNAs is based on the structural differences in their precursors, with siRNAs being formed from longer double-stranded RNA sequences [10,11]. In contrast to miRNAs, siRNA molecules are entirely complementary to mRNAs [12,13]. Small RNAs repress gene expression at the transcriptional or post-transcriptional level and have key functions in genome defense, growth, development, disease, and the stress response [14,15]. siRNAs regulate gene expression through transcriptional or post-transcriptional gene silencing [16,17]. They are crucial in regulating gene expression and are vital for plant development and responses to environmental stresses [18,19]. In contrast to miRNAs, which are evolutionarily highly conserved, siRNAs show a low degree of dependence on the dsRNA source [17,20]. To unambiguously determine the functions of both siRNAs and miRNAs, an analysis of the target genes regulated by the molecules is crucial. Experimental studies are needed to confirm the results obtained in the in silico analysis phase [9].

## 2. Small Interfering RNAs Biogenesis

Dicer-like (DCL) proteins are closely involved in siRNA biogenesis. DCL proteins can enzymatically cut double-stranded RNA (dsRNA) into short fragments [21,22]. Based on a structural analysis and consideration of domain structure differences, four classes of DCL proteins (DCL1, DCL2, DCL3, and DCL4) have been distinguished in model dicotyledonous plant species, namely *Arabidopsis thaliana* L. Additionally, each protein class has a distinct and specialized function [23,24]. DCL1 plays a pivotal role in the biogenesis of microRNAs (miRNAs), which serve as post-transcriptional regulators of gene expression in developmental and environmental adaptation processes [25]. DCL2 is responsible for processing double-stranded RNA (dsRNA) into 22-nucleotide small interfering RNAs (siRNAs) [26]. It plays a crucial role in antiviral defense and has functions similar to those of DCL4 in transgene silencing [27,28]. Additionally, DCL2 produces secondary siRNAs and transitive RNA silencing, enhancing other DCL proteins’ silencing efficiency [26,29]. DCL3 is a protein with a specific affinity for synthesizing 24-nucleotide siRNAs. This synthesis occurs in two steps: firstly, dsRNA is transcribed from heterochromatic loci using RNA polymerase IV, and secondly, the resulting dsRNA is converted into siRNA using RDR2 [26]. So, DCL3 is involved in the production of 24 nucleotide heterochromatic siRNAs (hc-siRNAs), and is involved in epigenetic regulation via RNA-directed DNA methylation (RdDM) [7,30,31]. This function is critical for maintaining genome stability and regulating gene expression at the transcriptional level [21]. DCL4 produces 21-nucleotide siRNAs, and is vital for the plant’s ability to respond to viral infections effectively. It cleaves long double-stranded RNAs into these smaller siRNAs, which can then guide the degradation of complementary mRNA targets, effectively silencing viral genes. Additionally, DCL4 is involved in the production of ta-siRNAs, which are crucial for regulating gene expression across different tissues [32,33,34]. PhasiRNA requires DCL2 and DCL4 for the vegetative phase change and disease resistance [3].

**Figure 2:**
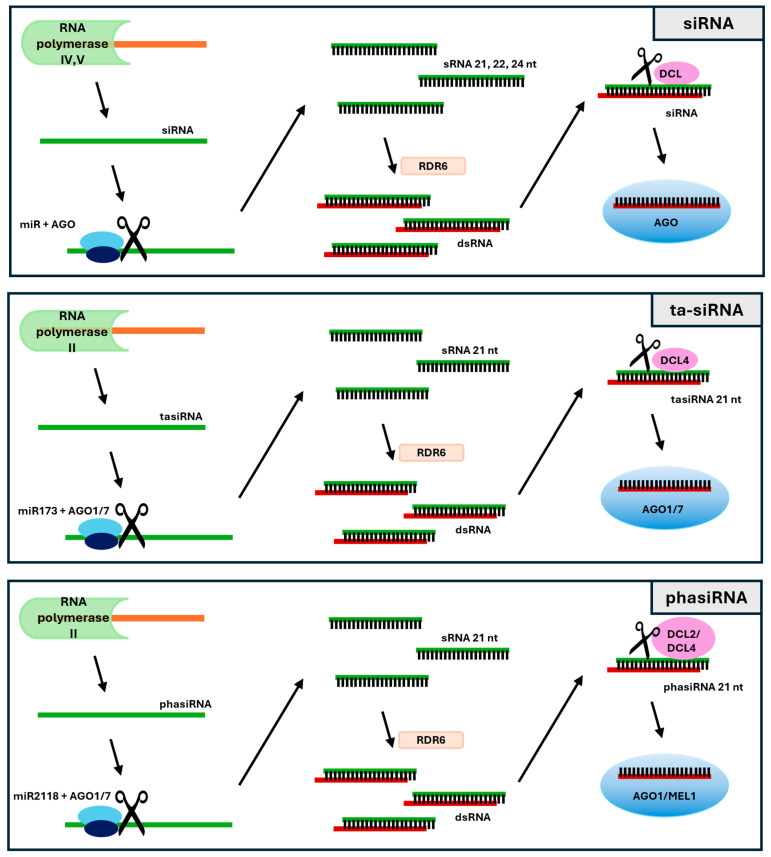
Biogenesis of siRNAs, ta-siRNAs and phasiRNAs in plants. Biogenesis involves the transcription of the *TAS* and *PHAS* loci, cleavage of primary transcripts involving miRNAs or AGO proteins, and the production of mature siRNAs incorporated into the RISC complex with AGO proteins.

Secondary siRNAs are produced from transcripts generated by RNA polymerase II in response to the activation of another miRNA or siRNA (Figure 2). The primary transcripts of secondary siRNAs are derived from *PHAS* or *TAS* loci. Precursors may be either coding transcripts or long non-coding RNAs. The origin of phasiRNAs can be traced back to genes from different protein-coding families. In contrast, lncRNAs serve as precursors for both ta-siRNAs and reproductive phasiRNAs. These sequences contain several twenty-nucleotide repeats [16,17]. In grasses, *PHAS* loci are mainly located in intergenic regions. In dicotyledonous plants (e.g., *A. thaliana*), 21 nt phasiRNAs are also generated from protein-coding genes [16].

Primary siRNA transcripts have a cap at the 5′ end and a poly(A) tail at the 3′ end. The first step in siRNA maturation is cutting the primary transcript to 22 nt with miRNA/AGO. During biogenesis, the presence of RdR2 polymerase is required. RdR2 recognizes the transcripts and uses them as a matrix to synthesize complementary RNA strands. As a result, multiple dsRNAs are formed from the original transcript. The DCL endonuclease trims the dsRNA into 21 nt ta-si/phasiRNA (DCL2/4) or 24 nt phasiRNA (DCL3b). Argonaute protein (AGO)-containing RISCs are incorporated into the duplex after one of the siRNA strands is degraded. The 21 nt ta-si/phasiRNAs are linked to AGO1. The AGO proteins that bind the 24 nt phasiRNA have yet to be fully understood, but recent studies suggest that they may be AGO2b and AGO18 [18]. This detailed process of siRNA biogenesis provides a comprehensive understanding of how these regulatory molecules are formed and function in plants.

Ta-siRNAs are a group of endogenous siRNAs that act in trans to regulate the expression of genes other than their own. The 21 nt long ta-siRNAs are generated from non-coding transcripts derived from *TAS* (trans-acting siRNA) genes by specific cleavage directed by miRNAs [35]. The cleaved ta-siRNA precursors are bound and stabilized by the gene silencing 3 (SGS3) suppressor and further synthesized into double-stranded RNAs by RDR6 [36,37]. Double-stranded RNAs are cleaved multiple times by DCL4 mediated by miRNAs. The resulting siRNA is a 21-nucleotide staged siRNA (ta-siRNA). Mature ta-siRNAs are incorporated into the AGO-RISC complex, cleaving target mRNAs or inhibiting translation [38,39]. Associated with the siRNA biogenesis process is the DCL ribonuclease, which acts by cleaving siRNA precursors into short duplexes that function as negative regulators of gene expression after binding to effector complex proteins and cleavage. Ta-siRNAs in complex with AGO1 or AGO7 proteins regulate the expression of target genes by inducing the truncation of their transcripts [40].

A specific group of plant siRNAs is the nat-siRNAs, derived from duplexes of RNA molecules that are synthesized in opposite directions on a matrix of one or two partially complementary loci [41,42]. The production of one of the transcripts is continuous, whereas the synthesis of the other transcript is induced by changing environmental conditions and pathogen infection [43]. The synthesized sequences form complementary ends of transcripts that are elongated by RdR6 polymerase and then cleaved into short dsRNAs with the participation of DCL1 and DCL2 [19].

## 3. Mechanisms of siRNA Activity

**Figure 3:**
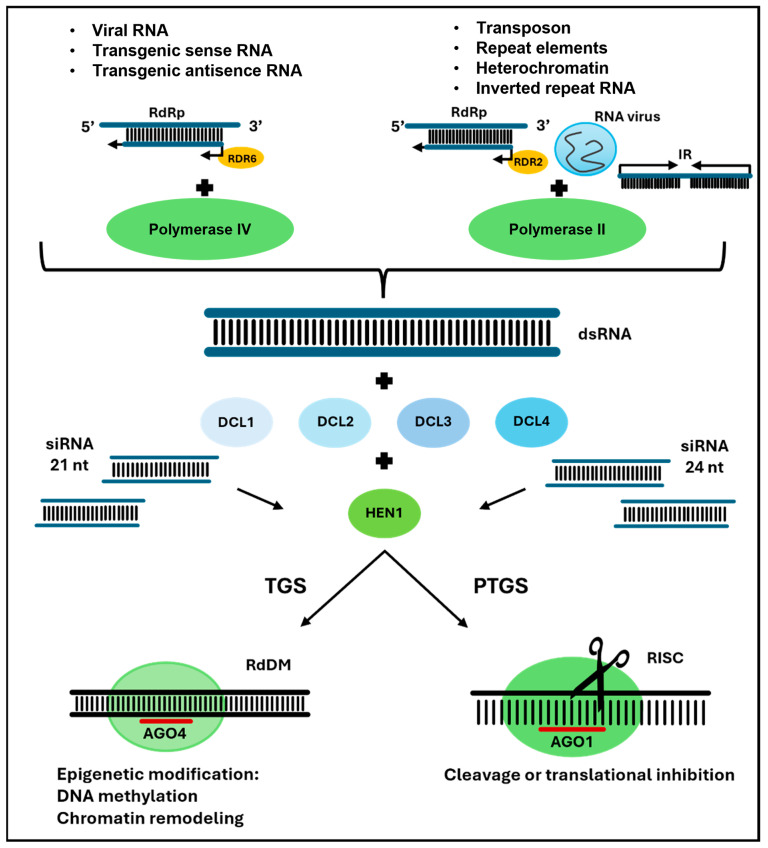
Mechanisms of the transcriptional gene silencing (TGS) or post-transcriptional gene silencing (PTGS) of siRNA-directed genes in plants.

mRNA silencing is an evolutionarily conserved pathway of sRNA sequence-specific gene interactions. Initially identified as an immune mechanism, it was later found to regulate growth stress responses and chromatin organization [44,45]. RNA silencing can occur via transcriptional gene silencing (TGS) or post-transcriptional gene silencing (PTGS). The active involvement of small interfering siRNAs was identified by analyzing the silencing pathways (Figure 3) [17]. Depending on their length, individual siRNA fragments have different functions at the mRNA level and in the epigenetic regulation of DNA (DNA methylation, histone modification, and gene silencing) [46]. The results of siRNA action are mRNA degradation, translational repression, histone methylation, or modification, depending on the effector complex’s protein composition and the target sequence’s nature [47,48].

The silencing of genes by PTGS results from mRNA degradation after transcription. The degradation of the mRNA results in the down-regulation of the proteins encoded by the cleaved transcripts. This process is initiated by a complementary siRNA of 21–24 nt. The siRNA molecule is incorporated into the RISC complex to initiate the cleavage process, which recognizes the target mRNA and prevents its translation [49]. The cleavage site is usually between the AGO protein’s 10th and 11th nucleotide positions. Although siRNAs are double-stranded, only one strand is loaded into the RISC silencing complex and can exert a silencing effect, similar to miRNAs. The thermodynamic stability of the 5’ ends of the duplex determines which strand is incorporated into RISC [50,51]. Histone modifications and DNA methylation mediate the TGS-based silencing mechanism. Twenty-four nt long siRNAs incorporated into AGO-RdDM protein complexes recognize complementary DNA sequences. The RdDM complex directs methyltransferases that methylate cytosines and lead to changes in chromatin structure. Histone modifications leading to the formation of a form of chromatin inaccessible to the transcriptional process also occur at the target sites of the sequences. Deposition of the active RISC complex within skeletal RNA recruits DRM2 methyltransferase, which initiates DNA sequence methylation [52,53]. AGO-siRNA complexes interact with RNA Pol V and its transcripts, initiating DNA methylation and participating in chromatin remodeling [19]. Sequences containing methylated cytosines are bound by enzymes involved in the epigenetic modification of histones (histone deacetylase, histone methyltransferases, K3K4-specific demethylase JUMONJI 14, and SWI/SNF family ATPase complexes). As a result of these epigenetic modifications, siRNAs mediate heterochromatinization of target DNA sequences [54].

RNA silencing was first discovered by Palaqui et al. in nitrate transgenic tobacco (*Nia gene*), where nitrogen inaccessibility and leaf chlorosis were the results [55]. The mobile nature of RNA silencing was first observed in the phloem sap of cucurbits, where it was proposed that all classes of siRNAs are capable of movement [56]. Plants use both canonical and non-canonical mechanisms of DNA methylation for epigenetic regulation. The non-canonical process, dependent on the RdDM machinery, uses siRNAs to direct de novo methylation, particularly of transposons and repetitive DNA. Switching between pathways occurs in response to siRNA levels and chromatin modifications, allowing plants to control gene expression through DNA methylation [43] dynamically.

## 4. Plant Development and Growth

During plant growth and development, the level of DNA methylation in various cell types and tissues is tightly controlled to prevent abnormal growth. PTGS and TGS mechanisms controlled by siRNAs counteract dynamic changes in gene expression levels [26]. The best-known mechanism of action of ta-siRNAs in *A. thaliana* is the regulation of somatic tissues [19]. On the other hand, in addition to the 24 nt siRNAs, the phasiRNAs play a specific role in the processes of sexual reproduction in plants. In monocotyledonous plants, reproductive 21 nt and 24 nt phasiRNAs play a particular role in this process [17]. Their roles are incompletely understood, but studies of maize and rice mutants indicate a critical role in male fertility [57]. In maize, the production of 21 nt phasiRNAs has been observed in other epidermal cells before meiotic entry. These phasiRNAs are bound by the AGO1 reproductive line-specific *MEL1* (meiosis arrested at leptotene 1). PhasiRNA levels decrease during further development. In contrast, tapetum cells synthesize 24 nt phasiRNAs during meiosis [58]. The movement of siRNAs after fertilization provides a unique opportunity for the maternal genome to respond to endosperm signals (e.g., growth and developmental rate) and potentially influence gene expression to maximize embryo success. It has been shown that siRNAs affect gene expression in *Brassica rapa* ovaries [59].

Both the 21 nt and 24 nt phasiRNAs are mobile and move toward the center of the anther after they are synthesized. Disorders such as excess number or an absence of meiocytes and abnormal tapetum differentiation result from the absence of phasiRNAs. The lack of differentiated microspore mother cells was mainly found in 24 nt phasiRNA mutants, indicating their crucial role in generative cell reprogramming. Wu et al. (2020), analyzing SKI2 and DCL4 mutants in *A. thaliana*, observed the involvement of 22 nt siRNAs in growth regulation and plant adaptation to environmental conditions. *A. thaliana* mutants with inhibited 22 nt siRNA synthesis showed the inhibition of root growth and delayed seedling development in soil [60]. The induction of 22 nt siRNA under drought stress caused translation slowing and inhibited plant growth. There are no conclusive studies to determine how 22 nt siRNAs mediate translational repression. It is speculated that the AGO1-22 nt siRNA complex interacts with ribosome function [60]. Through their interaction with miRNAs and hormone signaling in feedback loops, ta-siRNAs may be involved in seed maturation and germination. This is a relatively understudied aspect, and the molecular network of small RNA interactions affecting seed germination and dormancy remains a topic requiring further research [61]. In *Larix leptolepis*, the regulation of dormancy is associated with changes in the siRNA population [62]. Dormant embryos showed an increased accumulation of 24 nt siRNAs, whereas germinating seeds showed an increased presence of 21 nt siRNAs. The researchers suggested that the changes were related to the expression levels of the *RDR2* and *RDR6* genes [62]. In *A. thaliana*, the ta-siRNA-ARF module involves leaf morphology, developmental transformations, embryo development, responses to environmental stress, and flower and root morphology. Defects in TAS3-ta-siRNA biogenesis result in an accelerated transition to the generative plant stage and abnormal flower development [63]. TAS4-ta-siRNAs of *A. thaliana* cleave the transcripts of the *MYB-90, MYB-75*, and *MYB-113* genes and are thus involved in the anthocyanin accumulation pathway in leaves [29]. PhasiRNAs initiated by miR393 and miR3953 have been localized in auxin signaling pathways. They are involved in the transcription of *TIR* and *AFB2* genes in *Citrus* and *Litchi* to regulate flowering [64]. Studies in *A. thaliana* and rice indicate that subepidermal cells surrounding the tetrad produce 24 nt siRNAs that, in complex with AGO4-5, control female gametophyte development and differentiation [65]. miR390, identified in *A. thaliana* by the RNA induction of *TAS3*, initiates ta-siR-ARF formation [66,67]. The resulting siRNAs mediate the regulation of the transcript levels of the *ARF2*, *ARF3*, and *ARF4* genes, thereby regulating normal root development and leaf and flower formation [68,69]. The down-regulation of *ARF2* expression by siRNAs results in a delay in flowering, the senescence of rosette leaves, abscission of floral organs, and opening of siliques [70]. Glazinska et al. (2019) suggested in their study that the miR390 pathway, ta-siR-ARF, plays an essential role in plant development [67,71]. The regulation of *ARF2, ARF3,* and *ARF4* levels by ta-siR-ARF plays a vital role in developing lateral organs such as roots and leaves [68]. In *A. thaliana*, expression of the *TAS3* gene occurred in the upper part of the leaf. Ta-siR-ARF molecules exhibited the ability to move between cells, thus creating a gradient of their concentration in the lower part of the developing organ. This gradient was translated into the level of *ARF3* gene transcripts. The expression of miR390 was observed during the initiation of lateral root formation, which correlated with local ta-siRNA synthesis. The ta-siR-ARFs formed in the lateral root inhibited the activity of *ARF2*, *ARF3*, and *ARF4*, thus promoting this organ’s growth. The inhibition of ARF activity affected the average level of miR390 through a feedback loop [72]. Studies of the changes that occur in the embryonic shoot apical meristem (SAM) have shown that in *Oryza sativa*, the *SHL4/SHO2* gene (shootless 4/shoot organisation 2) encodes an *AGO7* ortholog, which is also found in *A. thaliana*. Mutations in this gene have been shown to affect leaf development through the ta-siRNA pathway. This pathway regulates a critical step in SAM formation during embryogenesis in *O. sativa*. Mutations in the *AGO5* gene in *Glycine max* resulted in the production of seeds with a saddle-shaped coloration pattern, and this effect affected the spatial distribution of siRNAs regulating the expression of the *CHS* (*chalcone synthase*) gene, which encodes chalcone synthase involved in the flavonoid synthesis pathway [23]. ARF2, ARF3, and ARF4 are down-regulated by ta-siRNA TAS3, which is involved in forming shoot apical meristems. TAS3 aggregation is regulated during embryogenesis in the SAM [73,74].

## 5. siRNA Network Mechanisms in the Stress Response

Small interfering RNAs play a crucial role in plant development, including regulating gene expression during environmental stress and defending plants against pathogens. Small interfering RNAs work by silencing specific genes, allowing plants to control growth and development processes and respond effectively to environmental stresses and viral infections. In addition, siRNAs protect the plant genome from the uncontrolled mobilization of genetic elements by playing an essential role in silencing transposons. As a result, siRNAs enhance the adaptability of plants, promoting their survival and genetic stability. The main mechanisms of siRNA action at the plant level are described below.

### 5.1. siRNA-Induced Response to Abiotic Stress

It has been hypothesized that the information accumulated during the lifetime of an organism is passed on to progeny via siRNAs that appear in response to environmental stimuli and form the so-called “environmental memory” or “plant immune memory,” which is transmitted epigenetically [75]. siRNAs play an essential role in the adaptation to environmental conditions by degrading or silencing transcripts that confer tolerance to abiotic stresses.

It has been observed that siRNAs identified in *Hordeum vulgare* control the response to drought stress during grain filling by targeting *HvCKX2.1* (*cytokinin oxidase 2.1*). Cytokinin oxidase was shown to be involved in cytokinin degradation and control of the germination time. During drought stress at the immature stage of the grain, there were increased production of 24 nt siRNAs and, increased levels of DNA methylation in the *HvCKX2.1* promoter region, thereby, affecting the rate of germination and shoot emergence through the accumulation of cytokinin ribosides [76]. In *Bruguiera gymnorrhiza*, TAS3-ta-siRNAs were found to be involved in the adaptation to stress conditions. In *H. vulgare* and *Triticum aestivum*, 24 nt phasiRNAs accumulate before the onset of meiosis, where AGO6 proteins probably bind them. These phasiRNAs maintain male fertility during environmental stress [77]. In their study, Borsani et al. (2005) observed a role for nat-siRNAs in the osmoprotection of *Arabidopsis* during salt and oxidative stress, which was related to the synthesis of nat-siRNAs from *SRO5* and *P5CDH* gene transcripts [78]. Under salt stress, a 24 nt nat-siRNA corresponding to the *SRO5* mRNA is produced. The synthesized 24 nt nat-siRNA degrades the *P5CDH* transcript, leading to the accumulation of proline and consequently increasing the tolerance of plants to salt stress [78]. Reduced P5CDH activity led to the accumulation of toxic P5C, which was counteracted by the SRO5 protein with detoxifying activity in the mitochondria [78]. The researchers demonstrated the involvement of the nat-siRNA SRO5-P5CDH as part of a regulatory loop that plays a role in the response to salt stress and the production of ROS [78]. The level of small non-coding RNAs was observed in *T. aestivum* seedlings during cold, heat, drought, and salinity stress. Changing expression levels of four siRNAs were observed: siRNA 005047_0654_1904. One was up-regulated by cold stress and down-regulated by heat, salinity, and drought; siRNA080621_1340_0098. One was up-regulated by cold and down-regulated by heat; siRNA002061_0636_3054. One was down-regulated by heat, excessive salinity, and drought stress, and siRNA007927_0100_2975.1 is down-regulated by salinity and cold drought stress [79]. *TAS1, TAS2,* and *TAS3* ta-siRNA transcripts showed increased expression in *A. thaliana* subjected to hypoxia [80]. *TAS* transcript levels were reflected by increases in miR-173 and miR-390 expression. The highly expressed miRNAs interacted with the pentatricopeptide repeat (PPR) proteins family. These proteins inhibit cytochromes and respiratory pathways in mitochondria [80]. However, in sweet potato, miR828 accumulates in wounded leaves, producing phasiRNA from its targets IbMYB and IbTLD, which function in cis to enhance their silencing. Finally, suppression of IbMYB and IbTLD increases lignin and hydrogen peroxide (H_2_O_2_) levels, protecting the plant from damage [81]. A siRNA has been identified in *Craterostigma plantagineum* that is induced during dehydration and may contribute to drought tolerance [82]. ABA induction and dehydration activate siRNA synthesis. The siRNA corresponds to the *CDT-1* locus (constitutively tolerating desiccation-1) [40]. The involvement of nat-siRNAATGB2 interacting with ETI (effector-induced immunity) via the *R* gene in response to environmental stress was observed by Katiyar et al. (2006). The induction of nat-siRNAATGB2 inhibits the expression of *PPRL* family genes, which are negative regulators of *ETI* [83]. siRNAs accumulate in response to infection with the *Heterodera schachtji* nematode; researchers observed a marked increase in the expression of siRNA9, siRNA41, and siRNA46 [84].

### 5.2. siRNA-Induced Response to Biotic Stress

Plants are constantly exposed to various infections (viruses, bacteria, and fungi) at every life cycle stage. In an immune response, plants have developed complex and precise defense mechanisms against pathogens [85,86]. siRNAs are primarily known for their role in silencing viral RNAs. However, the recent discovery of cross-species RNA interference (RNAi) has revealed an essential role for siRNAs in suppressing cellular pathogens. Recent evidence supports a novel mode of action for endogenous plant siRNAs, specifically in suppressing fungal infections by silencing pathogen genes. These studies suggest that siRNAs, typically composed of a mixture of different sequences, are used as a “shotgun” approach to target pathogen genes randomly but effectively. However, many aspects of cross-species RNAi still need to be clarified. The plant produces siRNAs that enter the fungal cells and silence the genes responsible for its virulence or ability to cause disease. This mechanism enables plants to defend themselves more effectively against fungal pathogens, reducing their abilities to cause infection and disease [87,88].

The first identified endogenous plant siRNA associated with biotic stress was nat-siRNA/ ATGB2, which regulates resistance gene-dependent effector-triggered immunity. This suggests that siRNAs may modulate the expression of pathogen-resistance genes [89]. The discovery of nat-siRNA/ATGB2 has been crucial in studying plant resistance to biotic stress. This siRNA is essential in regulating ETI-induced resistance, a plant defense mechanism activated by pathogen-effector proteins. The discovery suggests that siRNAs can not only silence the genetic material of foreign pathogens but also regulate the expression of genes in plants responsible for resistance to pathogens of different spectra, including bacteria, viruses, and fungi. Regulation by nat-siRNA/ATGB2 opens up new possibilities for modifying the resistance of all useful plants to increase their protection/tolerance to pathogens [89,90].

#### 5.2.1. siRNAs in Pathogen Infection

Studies have shown that the miRNA-phasiRNA pathway in plants can act as a regulatory center controlling growth and disease resistance. This includes controlling defense-related genes and mediating communication between the plant and the attacking pathogen. An example is research into disease resistance in legumes, where PHAS loci have been detected on mRNAs encoding several nucleotide-binding proteins with leucine-rich repeats (NBS-LRR) [91]. Studies have shown that sRNAs negatively regulate R NBS-LRR genes responsible for plant protection against pathogens [26]. The parasitic *Cuscuta*, which absorbs water and nutrients from mother plants, showed high levels of 22 nt miRNAs that target transcript degradation in *Arabidopsis* and *Nicotiana tabacum*, thereby affecting the production of secondary phasiRNAs. The phasiRNAs produced provide a mechanism for transgene regulation that allows the plant to adopt a parasitic lifestyle [92]. AGO7 in *Arabidopsis* has been shown to play an important role in plant–pathogen interactions. AGO7 mutants were found to be more susceptible to the fungal pathogen *Verticillium* [93]. AGO7 was observed to be involved in the symbiosis of rhizobia with legumes in *Lotus japonicas.* AGO7 is required for the biogenesis of TAS3 ta-siRNAs. These are involved in developing nitrogen-fixing papillae in plant roots [93]. The bacterial pathogen *Pseudomonas syringae* pv. *tomato* carrying the *AvrRpt2* effector strongly induces the plant nat-siRNAATGB2, which regulates plant immunity [83]. It has been observed that phasiRNAs control the expression of *NLR* genes. After pathogen infection, 22 nt miRNAs and phasiRNAs are downregulated. This leads to *NLR* regulation and subsequent activation of the immune response. The AGO1 protein is mainly associated with a 21-22 nt miRNA and a 5′-terminal uridine siRNA. *AGO1* loss-of-function mutants showed an impaired PTI response against bacterial pathogens in *A. thaliana* [94]. *RDR6* was shown to be essential for the activation of secondary siRNA production and the amplification of *shl2-rol* silencing signals. The *OsRDR6* mutant of rice showed enhanced necrosis after inoculation with *Xanthomonas oryzae* pv. *oryzae*, confirming the positive role of RDR6-dependent siRNAs in bacterial defense [95]. NB-LRRs are intracellular immune receptors. They recognize pathogen effectors and trigger plant immune responses. Without pathogens, NB-LRR transcripts are repressed by miRNA-triggered and RDR6-dependent secondary siRNAs [17]. In *Solanum lycopersicum*, the miR482/2118 family represses *NB-LRR*. Interference with miR482/2118 increased resistance to *P. syringae* [96]. Disruption of the miR472-RDR6 silencing pathway in *Arabidopsis*, which is required for *NB-LRR* gene repression, increased plant defense against *P. syringae* [97]. The *crp1 aba1* mutant of *Arabidopsis*, *SNC1,* an *R* protein, showed excessive accumulation in the nucleus. This led to a global reduction in NB-LRR-derived miRNAs and secondary siRNAs. This, in turn, increased resistance to *P. syringae* [98]. Transgenic barley and wheat expressing artificial siRNAs targeting the fungal effector gene Avra10 showed increased resistance to *Blumeria graminis*, an obligate biotrophic fungal pathogen that causes powdery mildew disease [99]. Plant sRNAs are transferred to invasive fungi, oomycetes, and parasitic plants, subsequently silencing parasite virulence genes and conferring immunity [2,100,101].

#### 5.2.2. siRNAs in Viral Infection

In 1999, viral siRNAs (vsiRNAs) were discovered in tobacco plants infected with the potato virus X [102]. Over the past two decades, RNAi has emerged as a promising and innovative tool in crop protection. It represents an exact and sustainable approach to controlling pests, pathogens, and other plant threats [103,104]. Antifungal activity was observed in studies on *Cucurbita pepo* L. and *Cucumis melo* L., where siRNA was used against the pathogen *B. cinerea* in the form of packed synthetic liposomes [105]. The vsiRNAs are derived from dsRNA precursors produced as intermediate replicative RNA virions or by bidirectional transcription of circular DNA virions [106]. Plant DCLs can directly recognize viral dsRNAs to produce primary siRNAs of 21–24 nt in length. To enhance the silencing signal, secondary siRNAs are processed by DCLs from long dsRNAs synthesized by RDR [102,107]. Secondary siRNAs can be loaded into AGOs to induce the degradation of single-stranded viral RNAs. Studies have shown that 21 nt siRNAs, mainly produced by DCL4, are the primary class of siRNAs that specifically silence viral RNAs via PTGS [26,108,109]. The critical importance of vsiRNAs for plant cells in degrading the viral genome and enhancing plant antiviral immunity has been observed. Some vsiRNAs can silence host gene expression or regulate host resistance to viral infection. The tomato yellow leaf curl virus (TYLCV) produces vsiRNAs by bidirectional RNA transcription from a short intergenic region. These vsiRNAs are used by TYLCV to silence SlLNR1. This gene is involved in antiviral defense in *S. lycopersicum* [110]. Another example is vsiRNAs derived from Wheat Yellow Mosaic Virus (WYMV). These vsiRNAs can activate broad-spectrum plant immunity by down-regulating host genes [111]. Transgenic *T. aestivum* expressing *vsiRNA1* showed increased resistance to viral infection. Further studies showed that vsiRNA1 silenced the *Triticum aestivum thioredoxin-like* (*TaAAED1*) gene, specifically to increase reactive oxygen species (ROS) production in a dose-dependent manner [111]. Another group of siRNAs involved in antiviral defense are virus-activated siRNAs (vasiRNAs), first identified in *Arabidopsis*. The vasiRNAs are mostly 21 nt long, genetically distinct from endogenous host siRNAs, and require DCL4 and RDR1 for their production. The vasiRNAs are generated from regions of host gene exons, although their role in modulating antiviral immunity remains unknown [112]. A recent study found that two *Brassica* species i.e., *Brassica rapa* and *Brassica napus*, also produce vasiRNAs [28]. The identified vasiRNAs interacted with target genes involved in photosynthesis and the stress response. Taken together, these studies suggest that they are involved in antiviral protection. Plant sRNAs, unbound and bound to RNA-binding proteins and encapsulated in vesicles, can move short distances through plasmodesmata, long distances through the phloem system, and even between species [113]. In tobacco, nat-miR6019 cleaves the tobacco mosaic virus resistance *N* gene [114]. The expression of some *NLR* and *PRR* family genes was suppressed by 22 nt miRNAs and secondary phasiRNAs in the absence of pathogens. This was performed to avoid autoimmune reactions and for energy consumption for plant growth [114].

## 6. Methods of siRNA Identification and Analysis

**Figure 4:**
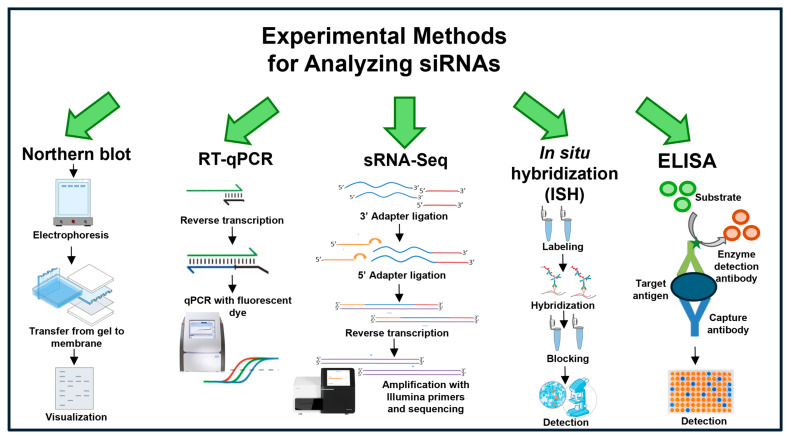
General experimental methods for analyzing siRNAs.

One method for siRNA detection is a pattern analysis based on Northern blot analysis, but the disadvantages of the presented technique are that it is time-consuming and has low sensitivity [115,116] (Figure 4). One of the most important steps is to obtain high-quality RNA from samples containing a fraction of sRNAs. The method involves the isolation of total RNA followed by electrophoretic separation in a polyacrylamide gel under denaturing conditions. Denaturation is achieved by adding urea, preventing secondary structure formation. The RNA is then transferred to a nitrocellulose or nylon membrane by electrotransfer or a capillary method. The siRNA sequences are hybridized to complementary probes on the membrane. The probes are either fluorescently or radioactively labeled, and the reaction conditions are controlled to match the thermodynamic properties of the probes. For detection, unbound probes are removed, and the image is developed on autoradiographic film or by fluorescence detection [115].

Another method used in siRNA research is RT-qPCR [117]. This method is used for the quantitative detection of siRNAs. It is one of the most sensitive methods and allows the analysis of siRNA expression levels even at deficient concentrations. The study is performed in several steps: isolation of RNA and reverse transcription to convert siRNAs into cDNAs. A critical step is the selection of sequence-specific primers due to the small size of siRNAs. The next step is the qPCR reaction. The amount of siRNA expressed is determined by detecting the fluorescence signal. This method has been used to study ta-siRNA and siRNAs in rice [117].

An alternative technique for detecting siRNAs in cells, tissues, and subcellular areas is in situ hybridization (ISH) [94]. The standard ISH method consists of tissue preparation by fixation, e.g., formaldehyde and embedding in paraffin. Choosing a process that protects the siRNAs from degradation is extremely important when performing siRNA fixation. Short probes designed as complementary sequences to the siRNAs are used for visualization. The probes are fluorescent or enzymatically labeled. The use of enzyme-labeled probes requires a subsequent staining and detection step. The prepared probes are then hybridized to the target siRNAs. It is essential to denature the probes to avoid double-stranded structures. Signals are detected by fluorescence or light microscopy to determine the exact location of the siRNAs. The method is beneficial for assessing the contributions of siRNAs to the responses to environmental stress and pathogen infection. It allows one to determine the location in the tissue where the siRNAs under investigation are activated [94].

An immunological method can be used to detect plant siRNAs. This method is based on the antigen–antibody ELISA (enzyme-linked immunosorbent assay) reaction [118]. The assay detects proteins and antigens and allows an indirect analysis of proteins typical of siRNA biosynthetic pathways such as DCL and AGO. Protein-specific antibodies bind to specific protein antigens, and then labeled secondary antibodies are added to generate a signal that is measured fluorescently or spectrophotometrically. This method was used by Shivaprasad et al. (2008) to investigate the role of AGO proteins in pathogen resistance.

Currently, the most widely used method for siRNA detection is sRNA-Seq [119]. The first step is isolating a fraction of small RNAs from plant material to which adaptors are ligated. The adapter-ligated RNA is reverse transcribed, followed by the amplification and final purification of NGS libraries. Sequencing is performed on the Illumina platform in the final step, generating reads of approximately 36 bp. The analysis allows for high sensitivity, generating millions of short reads in the fastQ file format [119]. A critical step in siRNA detection based on sRNA-Seq is to perform bioinformatic analyses. They allow the evaluation of the expression level and the prediction of the function of the identified siRNAs.

The identification of siRNAs by a bioinformatic analysis in plants is a subject that requires further development and standardization. The extant literature merely mentions the methods used in siRNA analyses, with no comprehensive methodology for handling this data type. Consequently, there is a necessity for the formulation of more precise and formalized guidelines for the execution of this analysis. The most significant challenge is to characterize those reads that may represent potential siRNAs and then predict their functions in the organism. Researchers have attempted to present different approaches to address this problem. Below are some selected approaches to siRNA identification.

A decade ago, Thiebaut et al. [120] decided to predict siRNA candidates in maize using the sRNA reads remaining after miRNA identification by the UEA sRNA Workbench. This protocol provides a comprehensive tool for siRNA analysis. The confirmation of sRNAs belonging to siRNAs was performed in two ways. The first was based on identifying siRNAs bound to repeats, while the second was linked to CDSs (coding DNA sequences).

In 2018, Mediana et al. proposed using the ShortStack tool to locate siRNA-containing loci. These were then used to search the coordinates to identify common clusters between the libraries. Only regions present in at least two of the three libraries were used for further expression analysis, allowing the location of differentially expressed clusters to be identified directly in the gene or at its predicted location [121].

Ge et al. and Ahmed et al. [122,123] performed siRNA identification based on two main criteria. For the sRNA reads obtained after the filtration, mapping, and removal of other structural RNA types, siRNA characterization was based on the number of a given read in the sample, which had to be greater than five, and the presence of two complementary overhanging bases. Ge et al. identified 861 differentially expressed siRNAs and 576 DE-siRNA target genes affecting callus development. In comparison, Ahmed et al. characterized 795 differentially expressed siRNA target genes for the Chinese cabbage heat stress response [122,123].

In 2022, Lei et al. presented an approach that uses the presence of NAT (natural antisense transcript) regions to identify siRNAs. Based on the assembled transcriptome, they set out to localize nat-siRNA fragments in soybeans. For this purpose, the NATpipe tool was used, which provides a ready-to-use protocol for the prediction of nat-siRNA loci and the search for phased sRNAs [124]. In addition to nat-siRNA predictions, sRNA reads were also screened for the presence of phasiRNAs using PhaseTank [125]. This allows the characterization of phasiRNAs and ta-siRNAs and provides comprehensive annotation for the identified genes [126]. Using this approach, the authors were able to detect a total of 26 novel siRNAs, including 17 phasiRNAs and 9 nat-siRNAs [125].

The 2023 article by Fu et al. performed siRNA identification without using additional criteria to confirm that a particular read belonged to the siRNA family. The focus was mainly on eliminating structural RNA from the filtered sRNA. The remaining reads were mapped to the wheat reference genome. This approach identified a total of 4019 siRNAs with a high level of confidence [127].

**Figure 5:**
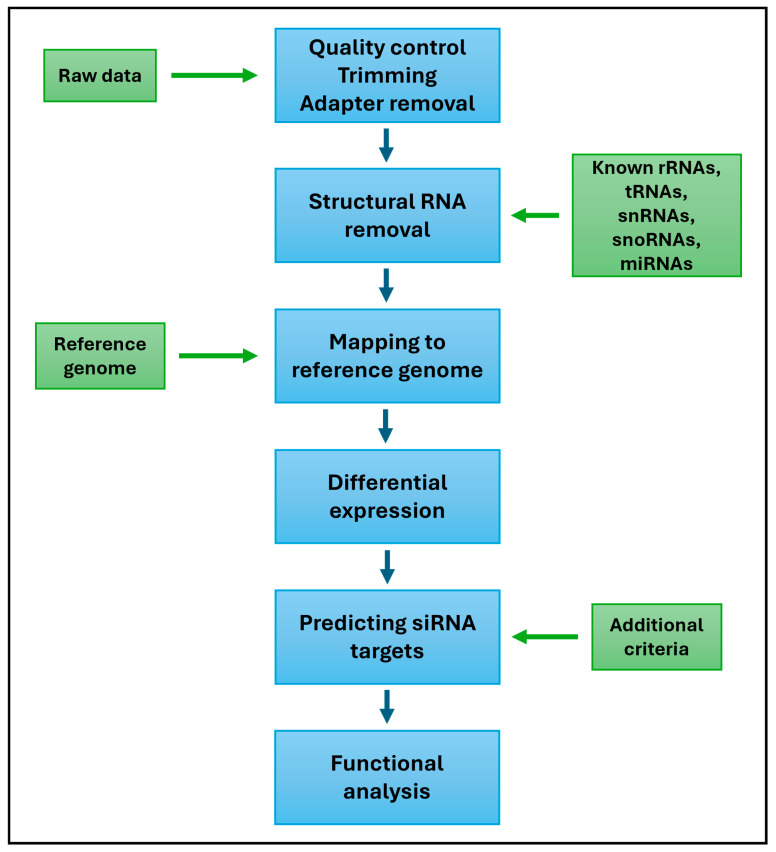
General protocol for siRNA identification.

The present review of the available literature confirms the diversity of approaches to siRNA identification in plants. The methods proposed by the authors differ in the steps, criteria, and bioinformatic tools used. However, a general protocol can be established based on these studies, which may be helpful in planning analyses related to siRNA identification (Figure 5).

The first step in the analysis is to check the quality of the reads obtained from sequencing. Illumina platforms are still subject to a specific error level; minimizing this before analyzing the data is critical for receiving reliable results. Therefore, quality control, removal of reads, and disposal of adapters should be performed first. All other structural RNAs (e.g., rRNA) are removed from data prepared this way. Available databases such as Rfam, RNACentral, and NCBI, from which known plant RNA family sequences can be extracted, will help with this. Once reads that do not belong to the sequences available in the databases have been identified, they can be mapped to a reference genome. The next step is to optionally analyze the differential expression of the identified siRNAs and predict target genes that may be affected by specific siRNAs. Several tools are available for siRNA target prediction. These include the psRobot tool, psRNATarget, and TargetFinder. The final step is to perform GO and KEGG enrichment analyses to determine which functions are affected by each identified siRNA.

## 7. Future Directions in Plant siRNA Research

As was outlined above, siRNAs represent critical components of post-transcriptional gene regulation in plants, playing pivotal roles in development, the stress response, and defense mechanisms. Recent advances in RNA interference research have expanded our knowledge of their biogenesis, molecular functions, and agricultural applications. However, several challenges and knowledge gaps remain.

siRNAs have a major impact on plant stress memory, a phenomenon whereby plants ‘remember’ previous stress events and respond more robustly to subsequent stress. Through mechanisms such as RNA-directed DNA methylation (RdDM) and chromatin remodeling, these molecules regulate stress-responsive genes [128,129]. A fundamental element to consider is the investigation of the longevity and reversibility of RNA-mediated epigenetic modifications across generations. Indeed, this is imperative for comprehending the potential of stress memory in long-term adaptation. Consequently, multi-generational studies of model plants and crops exposed to various stresses should be initiated. This type of analysis is essential to ascertain the duration of such changes in stress memory. Nonetheless, research involving non-model species that exhibit longer life cycles may be impeded by the prevailing paradigm of short-term research funding (typically 3–5 years), as it is crucial to identify factors that facilitate the reversal of epigenetic modifications. This will enhance our comprehension of the mechanisms that regulate the transmission of stress memory from one generation to the next, paving the way for the development strategies to augment the stability of beneficial epigenetic modifications in plant breeding programs. It is also important to consider the potential for further exploration into the temporal dynamic interactions between methylation and histone modifications mediated by siRNAs. This could facilitate a comprehensive understanding of stress memory maintenance. However, it is necessary to develop computational models to predict the interactions between different epigenetic marks in stress memory formation to achieve this. Consequently, this could result in the availability of arrays of epigenetic editing tools to manipulate stress memory in crops. Considering the points above, it is evident that further detailed profiling of stress-specific siRNAs is of significant importance. Such detailed profiling is crucial to facilitate an understanding of how plants adapt their responses to different types and intensities of stress. One potential outcome of this type of research could be the development of atlases of stress-specific siRNAs for major crop species. Furthermore, such information could be translated into developing diagnostic tools to assess the plant stress status based on siRNA profiles. To provide a more complete picture of the regulatory networks of plant responses to stress and the formation and maintenance of stress memory, further knowledge of the interactions between siRNAs and other non-coding RNAs is required. This can be achieved by conducting integrated analyses of siRNAs, and miRNAs, lncRNA expression patterns, and transcription factors. This approach offers the potential to identify novel RNA-based mechanisms that fine-tune plants’ stress responses and develop RNA-based strategies to enhance plant resistance to stress. Undoubtedly, the ultimate goal of such research is to develop strategies to use siRNA-mediated stress memory to enhance crop resilience and productivity. Both collections clustered in gene banks, which allow testing for natural variation in the siRNA-mediated stress memory capacity, and modern genetic engineering and gene editing techniques to enhance siRNA production or stability in stress-responsive pathways can play a significant role in it. This work could be of great utilitarian importance to the human species in an era of intense climate change. It is conceivable that, due to the knowledge accumulated during basic research, new crop varieties with enhanced stress memory and increased resistance to multiple stresses will be developed, or new agronomic practices that exploit siRNA-mediated stress memory for sustainable agriculture will be developed. By pursuing these lines of research, scientists can greatly expand our knowledge of siRNA-mediated stress memory in plants. This knowledge will be essential for developing innovative approaches to improve plant stress tolerance and yield, meet the challenges of changing environmental conditions, and ensure global food security. The utilization of siRNAs in the domain of crop improvement represents a rapidly expanding area of the research endeavor. This technological advancement has elicited resistance to viral, bacterial, and fungal pathogens and augmented tolerance to abiotic stresses such as drought and salinity [130,131]. Developing environmentally friendly delivery systems for siRNAs, such as those for spraying or soil treatments, is crucial for enhancing the accessibility and practicality of this technology for large-scale agricultural applications. In this regard, it is vital to explore new nanoparticle formulations to enhance plant stability and cellular uptake. In addition, the potential of using beneficial microorganisms as vectors for siRNA delivery should be explored. Developing environmentally friendly, cost-effective delivery methods for field applications and integrated pest management strategies using siRNA technology would be a significant achievement.

## 8. Conclusions

Small interfering RNAs (siRNAs) were discovered in the nematode *Caenorhabditis elegans*, whose genome was sequenced in 1998 [132]. The discovery of siRNAs in nematodes was a breakthrough in molecular biology and next-generation sequencing. siRNAs are short RNA molecules that play a vital role in the plant’s defense responses to pathogens and environmental stress. Plants use siRNAs to inhibit or degrade the expression of target transcripts in defense mechanisms based on the process of RNA interference (RNAi). They have different functions in different biological processes, such as regulating the expression of genes involved in growth, development, and the plant’s response to a stress factor (abiotic or biotic), depending on their function in the plant genome.

In recent years, studies have significantly increasingly focused on the roles of siRNAs and their influence on plant developmental processes. This growing body of research opens new perspectives for developing biotechnology and modern, sustainable plant breeding. This development has allowed for a better understanding of the roles of miRNAs and siRNAs in regulating plant development at the mRNA level, mainly through gene silencing and the regulation of mRNA degradation.

Studies in recent years have shown that the action of sRNAs is often tightly linked to hormonal regulation in plants, primarily by affecting the tissue and temporal localization of elements of hormonal pathways. Enormous advances in the understanding of siRNA action have been brought about by the knowledge that siRNAs can move between cells and create a concentration gradient similar to that of hormones. In addition, sRNAs are often “linkers” between the actions of different phytohormones [133].

A fundamental discovery for modern resistance breeding is RNA interference. The ability to precisely silence specific genes from viral, fungal, and other dangerous pathogens could be the foundation for sustainable agriculture in the future. In this era of climate change, using siRNAs as a biotechnological tool in modern breeding seems particularly relevant. The regulation of genes responsible for abiotic stress tolerance using the siRNA mechanism is crucial for the survival and acclimatization of plants under extreme weather conditions such as prolonged drought, high temperature, and rapid rainfall. In conclusion, siRNA research is crucial for agriculture’s future and food security maintenance.

Research on siRNAs in plants has made significant progress. However, there are still many unanswered questions. An important direction is the functional characterization of new classes of siRNAs and the role of siRNAs in interspecies interactions, such as communication between plants and microbes. An area requiring further research is the involvement of siRNAs in regulating gene expression through DNA methylation and epigenetic modifications of DNA. The knowledge of siRNAs can contribute to improving crop plants through precise gene regulation. siRNAs can enhance pathogen resistance and tolerance to abiotic stresses. Analyzing the conservation of siRNA pathways in different species and studying their co-evolution with target sequences will provide valuable information on plant adaptations to changing environmental conditions. Solving these problems will bring new opportunities for precision agriculture, ecology, medicine, and pharmaceuticals.

## Figures and Tables

**Table 1:** The differences and similarities between siRNAs and miRNAs.

Category	siRNAs	miRNAs
Origins	Encoded by transposons, virus, heterochromatin	Distinct genomic loci encoded by their genes
Biogenesis, precursor	Long bimolecular RNA	sRNA molecules that include an imperfect stem-loop secondary structure
Processing enzyme	Dicer-like enzymes	
Accessory proteins	Different cofactors depending on the siRNA class, e.g., RDRs	SE and HYL1 proteins
Length	21, 23, 24 nt	18–24 nt
Mechanism of action	Associate with RISC to mediate mRNA TGS and PTGS mechanisms	Mediate the silencing of the same genes from which they originate
Working principle	Function through RISC by complementarity targeting mRNA	
Complementarity to targets	Fully complementary to mRNA	Partially complementary to the mRNA targets
Sequence conservation	Low conservation, depending on the dsRNA source	Highly conserved across plant species
Epigenetic effects	Induce TGS via DNA methylation	Typically absent

## Data Availability

No new data were created or analyzed in this study.

## References

[1] An Y., Su H., Niu Q., Yin S. (2022). Integrated Analysis of Coding and Non-coding RNAs Reveals the Molecular Mechanism Underlying Salt Stress Response in Medicago truncatula. Front. Plant Sci..

[2] Cao W., Yang L., Zhuang M., Lv H., Wang Y., Zhang Y., Ji J. (2024). Plant non-coding RNAs: The new frontier for the regulation of plant development and adaptation to stress. Plant Physiol. Biochem..

[3] Liu H., Able A.J., Able J.A. (2020). Integrated Analysis of Small RNA, Transcriptome, and Degradome Sequencing Reveals the Water-Deficit and Heat Stress Response Network in Durum Wheat. Int. J. Mol. Sci..

[4] Zhang P., Wu W., Chen Q., Chen M. (2019). Non-Coding RNAs and their Integrated Networks. J. Integr. Bioinform..

[5] Wang Y., Deng X.W., Zhu D. (2022). From molecular basics to agronomic benefits: Insights into noncoding RNA-mediated gene regulation in plants. J. Integr. Plant Biol..

[6] Sun M. (2025). Advances in Plant Epigenetic Regulation of Abiotic Stress Response. Theor. Nat. Sci..

[7] Fei Q., Xia R., Meyers B.C. (2013). Phased, Secondary, Small Interfering RNAs in Posttranscriptional Regulatory Networks. Plant Cell.

[8] Tang G. (2005). siRNA and miRNA: An insight into RISCs. Trends Biochem. Sci..

[9] Miskiewicz J., Tomczyk K., Mickiewicz A., Sarzynska J., Szachniuk M. (2017). Bioinformatics Study of Structural Patterns in Plant MicroRNA Precursors. BioMed Res. Int..

[10] Carthew R.W., Sontheimer E.J. (2009). Origins and Mechanisms of miRNAs and siRNAs. Cell.

[11] Mattick J.S., Amaral P.P., Carninci P., Carpenter S., Chang H.Y., Chen L.-L., Chen R., Dean C., Dinger M.E., Fitzgerald K.A. (2023). Long non-coding RNAs: Definitions, functions, challenges and recommendations. Nat. Rev. Mol. Cell Biol..

[12] Lam J.K.W., Chow M.Y.T., Zhang Y., Leung S.W.S. (2015). siRNA Versus miRNA as Therapeutics for Gene Silencing. Mol. Ther. Nucleic Acids.

[13] Ranasinghe P., Addison M.L., Dear J.W., Webb D.J. (2023). Small interfering RNA: Discovery, pharmacology and clinical development—An introductory review. Br. J. Pharmacol..

[14] Zhu J.-K. (2008). Reconstituting plant miRNA biogenesis. Proc. Natl. Acad. Sci. USA.

[15] Yang S., Kim S.-H., Yang E., Kang M., Joo J.-Y. (2024). Molecular insights into regulatory RNAs in the cellular machinery. Exp. Mol. Med..

[16] Filipowicz W., Jaskiewicz L., Kolb F.A., Pillai R.S. (2005). Post-transcriptional gene silencing by siRNAs and miRNAs. Curr. Opin. Struct. Biol..

[17] Vaucheret H., Voinnet O. (2023). The plant siRNA landscape. Plant Cell.

[18] Phillips J.R., Dalmay T., Bartels D. (2007). The role of small RNAs in abiotic stress. FEBS Lett..

[19] Zhan J., Meyers B.C. (2023). Plant Small RNAs: Their Biogenesis, Regulatory Roles, and Functions. Annu. Rev. Plant Biol..

[20] Bielewicz D., Kalak M., Kalyna M., Windels D., Barta A., Vazquez F., Szweykowska-Kulinska Z., Jarmolowski A. (2013). Introns of plant pri-miRNAs enhance miRNA biogenesis. Embo Rep..

[21] Qin C., Li B., Fan Y., Zhang X., Yu Z., Ryabov E., Zhao M., Wang H., Shi N., Zhang P. (2017). Roles of Dicer-Like Proteins 2 and 4 in Intra- and Intercellular Antiviral Silencing. Plant Physiol..

[22] Wang Q., Xue Y., Zhang L., Zhong Z., Feng S., Wang C., Xiao L., Yang Z., Harris C.J., Wu Z. (2021). Mechanism of siRNA production by a plant Dicer-RNA complex in dicing-competent conformation. Science.

[23] Liu Q., Feng Y., Zhu Z. (2009). Dicer-like (DCL) proteins in plants. Funct. Integr. Genom..

[24] Nielsen C.P.S., Arribas-Hernández L., Han L., Reichel M., Woessmann J., Daucke R., Bressendorff S., López-Márquez D., Andersen S.U., Pumplin N. (2024). Evidence for an RNAi-independent role of Arabidopsis DICER-LIKE2 in growth inhibition and basal antiviral resistance. Plant Cell.

[25] Akbar S., Wei Y., Zhang M.-Q. (2022). RNA Interference: Promising Approach to Combat Plant Viruses. Int. J. Mol. Sci..

[26] Sanan-Mishra N., Jailani A.A.K., Mandal B., Mukherjee S.K. (2021). Secondary siRNAs in Plants: Biosynthesis, Various Functions, and Applications in Virology. Front. Plant Sci..

[27] Parent J., Bouteiller N., Elmayan T., Vaucheret H. (2014). Respective contributions of Arabidopsis DCL2 and DCL4 to RNA silencing. Plant J..

[28] Leonetti P., Ghasemzadeh A., Consiglio A., Gursinsky T., Behrens S., Pantaleo V. (2020). Endogenous activated small interfering RNAs in virus-infected Brassicaceae crops show a common host gene-silencing pattern affecting photosynthesis and stress response. New Phytol..

[29] Mlotshwa S., Pruss G.J., Peragine A., Endres M.W., Li J., Chen X., Poethig R.S., Bowman L.H., Vance V. (2008). DICER-LIKE2 Plays a Primary Role in Transitive Silencing of Transgenes in Arabidopsis. PLoS ONE.

[30] Holoch D., Moazed D. (2015). RNA-mediated epigenetic regulation of gene expression. Nat. Rev. Genet..

[31] Hamar É., Szaker H.M., Kis A., Dalmadi Á., Miloro F., Szittya G., Taller J., Gyula P., Csorba T., Havelda Z. (2020). Genome-Wide Identification of RNA Silencing-Related Genes and Their Expressional Analysis in Response to Heat Stress in Barley (*Hordeum vulgare* L.). Biomolecules.

[32] Xie Z., Allen E., Wilken A., Carrington J.C. (2005). DICER-LIKE 4 functions in trans-acting small interfering RNA biogenesis and vegetative phase change in *Arabidopsis thaliana*. Proc. Natl. Acad. Sci. USA.

[33] Kakiyama S., Tabara M., Nishibori Y., Moriyama H., Fukuhara T. (2019). Long DCL4-substrate dsRNAs efficiently induce RNA interference in plant cells. Sci. Rep..

[34] Montavon T., Kwon Y., Zimmermann A., Hammann P., Vincent T., Cognat V., Bergdoll M., Michel F., Dunoyer P. (2018). Characterization of DCL4 missense alleles provides insights into its ability to process distinct classes of dsRNA substrates. Plant J..

[35] Devers E.A., Brosnan C.A., Sarazin A., Schott G., Lim P., Lehesranta S., Helariutta Y., Voinnet O. (2023). *In planta* dynamics, transport biases, and endogenous functions of mobile siRNAs in *Arabidopsis*. Plant J..

[36] Chen X. (2009). Small RNAs and Their Roles in Plant Development. Annu. Rev. Cell Dev. Biol..

[37] Halder K., Chaudhuri A., Abdin M.Z., Datta A. (2023). Tweaking the Small Non-Coding RNAs to Improve Desirable Traits in Plant. Int. J. Mol. Sci..

[38] Allen E., Xie Z., Gustafson A.M., Carrington J.C. (2005). microRNA-Directed Phasing during Trans-Acting siRNA Biogenesis in Plants. Cell.

[39] Kunej U., Jakše J., Radišek S., Štajner N. (2021). Core RNA interference genes involved in miRNA and ta-siRNA biogenesis in hops and their expression analysis after challenging with verticillium nonalfalfae. Int. J. Mol. Sci..

[40] Chen X., Rechavi O. (2021). Plant and animal small RNA communications between cells and organisms. Nat. Rev. Mol. Cell Biol..

[41] Hollick J.B. (2012). Paramutation: A trans-homolog interaction affecting heritable gene regulation. Curr. Opin. Plant Biol..

[42] Tang Y., Yan X., Gu C., Yuan X. (2022). Biogenesis, Trafficking, and Function of Small RNAs in Plants. Front. Plant Sci..

[43] Pikaard C.S., Haag J.R., Ream T., Wierzbicki A.T. (2008). Roles of RNA polymerase IV in gene silencing. Trends Plant Sci..

[44] Shabalina S., Koonin E. (2008). Origins and evolution of eukaryotic RNA interference. Trends Ecol. Evol..

[45] Kryovrysanaki N., James A., Tselika M., Bardani E., Kalantidis K. (2022). RNA silencing pathways in plant development and defense. Int. J. Dev. Biol..

[46] Baumann K. (2020). How plants silence stress. Nat. Rev. Mol. Cell Biol..

[47] Guleria P., Mahajan M., Bhardwaj J., Yadav S.K. (2011). Plant Small RNAs: Biogenesis, Mode of Action and Their Roles in Abiotic Stresses. Genom. Proteom. Bioinform..

[48] Dana H., Chalbatani G.M., Mahmoodzadeh H., Gharagouzlo E., Karimloo R., Rezaiean O., Moradzadeh A., Mazraeh A., Marmari V., Rashno M.M. (2017). Molecular Mechanisms and Biological Functions of siRNA. Int. J. Biomed. Sci..

[49] Orban T.I., Izaurralde E. (2005). Decay of mRNAs targeted by RISC requires XRN1, the Ski complex, and the exosome. RNA.

[50] Tomari Y., Zamore P.D. (2005). Perspective: Machines for RNAi. Genes Dev..

[51] Liu W., Shoji K., Naganuma M., Tomari Y., Iwakawa H.-O. (2022). The mechanisms of siRNA selection by plant Argonaute proteins triggering DNA methylation. Nucleic Acids Res..

[52] Wierzbicki A.T., Cocklin R., Mayampurath A., Lister R., Rowley M.J., Gregory B.D., Ecker J.R., Tang H., Pikaard C.S. (2012). Spatial and functional relationships among Pol V-associated loci, Pol IV-dependent siRNAs, and cytosine methylation in the *Arabidopsis* epigenome. Genes Dev..

[53] Erdmann R.M., Picard C.L. (2020). RNA-directed DNA methylation. PLoS Genet..

[54] Ashapkin V.V., Kutueva L.I., Aleksandrushkina N.I., Vanyushin B.F. (2019). Epigenetic Regulation of Plant Gametophyte Development. Int. J. Mol. Sci..

[55] Palauqui J.C., Elmayan T., de Borne F.D., Crete P., Charles C., Vaucheret H. (1996). Frequencies, Timing, and Spatial Patterns of Co-Suppression of Nitrate Reductase and Nitrite Reductase in Transgenic Tobacco Plants. Plant Physiol..

[56] Yoo B.-C., Kragler F., Varkonyi-Gasic E., Haywood V., Archer-Evans S., Lee Y.M., Lough T.J., Lucas W.J. (2004). A Systemic Small RNA Signaling System in Plants. Plant Cell.

[57] Zhai J., Zhang H., Arikit S., Huang K., Nan G.-L., Walbot V., Meyers B.C. (2015). Spatiotemporally dynamic, cell-type–dependent premeiotic and meiotic phasiRNAs in maize anthers. Proc. Natl. Acad. Sci. USA.

[58] Zhang Y.-C., Lei M.-Q., Zhou Y.-F., Yang Y.-W., Lian J.-P., Feng Y.-Z., Zhou K.-R., He R.-R., He H., Zhang Z. (2020). Reproductive phasiRNAs regulate reprogramming of gene expression and meiotic progression in rice. Nat. Commun..

[59] Chow H.T., Mosher R.A. (2023). Small RNA-mediated DNA methylation during plant reproduction. Plant Cell.

[60] Wu H., Li B., Iwakawa H.-O., Pan Y., Tang X., Ling-Hu Q., Liu Y., Sheng S., Feng L., Zhang H. (2020). Plant 22-nt siRNAs mediate translational repression and stress adaptation. Nature.

[61] Wu J., Huang W., He Z. (2013). Dendrimers as Carriers for siRNA Delivery and Gene Silencing: A Review. Sci. World J..

[62] Zhang J., Zhang S., Han S., Li X., Tong Z., Qi L. (2013). Deciphering Small Noncoding RNAs during the Transition from Dormant Embryo to Germinated Embryo in Larches (Larix leptolepis). PLoS ONE.

[63] Chitwood D.H., Nogueira F.T., Howell M.D., Montgomery T.A., Carrington J.C., Timmermans M.C. (2009). Pattern formation via small RNA mobility. Genes Dev..

[64] Liu Y., Ke L., Wu G., Xu Y., Wu X., Xia R., Deng X., Xu Q. (2017). miR3954 is a trigger of phasiRNAs that affects flowering time in citrus. Plant J..

[65] Tucker M., Okada T., Hu Y., Scholefield A., Taylor J.M., Koltunow A.M.G. (2012). Somatic small RNA pathways promote the mitotic events of megagametogenesis during female reproductive development in Arabidopsis. Development.

[66] Montgomery T.A., Howell M.D., Cuperus J.T., Li D., Hansen J.E., Alexander A.L., Chapman E.J., Fahlgren N., Allen E., Carrington J.C. (2008). Specificity of ARGONAUTE7-miR390 Interaction and Dual Functionality in TAS3 Trans-Acting siRNA Formation. Cell.

[67] Xia R., Xu J., Meyers B.C. (2017). The emergence, evolution, and diversification of the miR390-TAS3-ARF pathway in land plants. Plant Cell.

[68] Hunter C., Willmann M.R., Wu G., Yoshikawa M., de la Luz Gutiérrez-Nava M., Poethig S.R. (2006). Trans-acting siRNA-mediated repression of ETTIN and ARF4 regulates heteroblasty in Arabidopsis. Development.

[69] Luo L., Yang X., Guo M., Lan T., Yu Y., Mo B., Chen X., Gao L., Liu L. (2021). *TRANS-ACTING SIRNA3*-derived short interfering RNAs confer cleavage of mRNAs in rice. Plant Physiol..

[70] Ellis C.M., Nagpal P., Young J.C., Hagen G., Guilfoyle T.J., Reed J.W. (2005). *AUXIN RESPONSE FACTOR1* and *AUXIN RESPONSE FACTOR2* regulate senescence and floral organ abscission in *Arabidopsis thaliana*. Development.

[71] Glazińska P., Kulasek M., Glinkowski W., Wojciechowski W., Kosiński J. (2019). Integrated Analysis of Small RNA, Transcriptome and Degradome Sequencing Provides New Insights into Floral Development and Abscission in Yellow Lupine (*Lupinus luteus* L.). Int. J. Mol. Sci..

[72] Marin E., Jouannet V., Herz A., Lokerse A.S., Weijers D., Vaucheret H., Nussaume L., Crespi M.D., Maizel A. (2010). miR390, Arabidopsis TAS3 tasiRNAs, and Their AUXIN RESPONSE FACTOR Targets Define an Autoregulatory Network Quantitatively Regulating Lateral Root Growth. Plant Cell.

[73] Alhassan A.R., Dzinyela R., Dargiri S.A., Suglo P., Yang L., Movahedi A. (2025). Regulatory roles of small RNAs in plant growth, breeding, and stress adaptation. Plant Growth Regul..

[74] Kirolinko C., Hobecker K., Wen J., Mysore K.S., Niebel A., Blanco F.A., Zanetti M.E. (2021). Auxin Response Factor 2 (ARF2), ARF3, and ARF4 Mediate Both Lateral Root and Nitrogen Fixing Nodule Development in Medicago truncatula. Front. Plant Sci..

[75] Molinier J., Ries G., Zipfel C., Hohn B. (2006). Transgeneration memory of stress in plants. Nature.

[76] Surdonja K., Eggert K., Hajirezaei M.-R., Harshavardhan V.T., Seiler C., Von Wirén N., Sreenivasulu N., Kuhlmann M. (2017). Increase of DNA Methylation at the *HvCKX2.1* Promoter by Terminal Drought Stress in Barley. Epigenomes.

[77] Bélanger S., Pokhrel S., Czymmek K.J., Meyers B.C. (2020). Pre-meiotic, 24-nt reproductive phasiRNAs are abundant in anthers of wheat and barley but not rice and maize. Plant Physiol..

[78] Borsani O., Zhu J., Verslues P.E., Sunkar R., Zhu J.-K. (2005). Endogenous siRNAs Derived from a Pair of Natural cis-Antisense Transcripts Regulate Salt Tolerance in Arabidopsis. Cell.

[79] Yao Y., Ni Z., Peng H., Sun F., Xin M., Sunkar R., Zhu J.-K., Sun Q. (2010). Non-coding small RNAs responsive to abiotic stress in wheat (Triticum aestivum L.). Funct. Integr. Genom..

[80] Moldovan D., Spriggs A., Yang J., Pogson B.J., Dennis E.S., Wilson I.W. (2009). Hypoxia-responsive microRNAs and trans-acting small interfering RNAs in Arabidopsis. J. Exp. Bot..

[81] Liu Y., Teng C., Xia R., Meyers B.C. (2020). PhasiRNAs in Plants: Their Biogenesis, Genic Sources, and Roles in Stress Responses, Development, and Reproduction. Plant Cell.

[82] Hilbricht T., Varotto S., Sgaramella V., Bartels D., Salamini F., Furini A. (2008). Retrotransposons and siRNA have a role in the evolution of desiccation tolerance leading to resurrection of the plant *Craterostigma plantagineum*. New Phytol..

[83] Katiyar-Agarwal S., Morgan R., Dahlbeck D., Borsani O., Villegas A., Zhu J.-K., Staskawicz B.J., Jin H. (2006). A pathogen-inducible endogenous siRNA in plant immunity. Proc. Natl. Acad. Sci. USA.

[84] Hewezi T., Howe P., Maier T.R., Baum T.J. (2008). *Arabidopsis* Small RNAs and Their Targets During Cyst Nematode Parasitism. Mol. Plant-Microbe Interact..

[85] Jones J.D.G., Dangl J.L. (2006). The plant immune system. Nature.

[86] Dodds P.N., Chen J., Outram M.A. (2024). Pathogen perception and signaling in plant immunity. Plant Cell.

[87] Qiao Y., Xia R., Zhai J., Hou Y., Feng L., Zhai Y., Ma W. (2021). Small RNAs in Plant Immunity and Virulence of Filamentous Pathogens. Annu. Rev. Phytopathol..

[88] Mann C.W., Sawyer A., Gardiner D.M., Mitter N., Carroll B.J., Eamens A.L. (2023). RNA-based control of fungal pathogens in plants. Int. J. Mol. Sci..

[89] Khraiwesh B., Zhu J.-K., Zhu J. (2012). Role of miRNAs and siRNAs in biotic and abiotic stress responses of plants. Biochim. Biophys. Acta.

[90] Ding T., Li W., Li F., Ren M., Wang W. (2024). microRNAs: Key Regulators in Plant Responses to Abiotic and Biotic Stresses via Endogenous and Cross-Kingdom Mechanisms. Int. J. Mol. Sci..

[91] Jyothsna S., Alagu M. (2022). Role of phasiRNAs in plant-pathogen interactions: Molecular perspectives and bioinformatics tools. Physiol. Mol. Biol. Plants.

[92] Shahid S., Kim G., Johnson N.R., Wafula E., Wang F., Coruh C., Bernal-Galeano V., Phifer T., Depamphilis C.W., Westwood J.H. (2018). MicroRNAs from the parasitic plant Cuscuta campestris target host messenger RNAs. Nature.

[93] Ellendorff U., Fradin E.F., de Jonge R., Thomma B.P.H.J. (2008). RNA silencing is required for Arabidopsis defence against Verticillium wilt disease. J. Exp. Bot..

[94] Li Y., Zhang Q., Zhang J., Wu L., Qi Y., Zhou J.-M. (2010). Identification of MicroRNAs Involved in Pathogen-Associated Molecular Pattern-Triggered Plant Innate Immunity. Plant Physiol..

[95] Wagh S.G., Alam M.M., Kobayashi K., Yaeno T., Yamaoka N., Toriba T., Hirano H.-Y., Nishiguchi M. (2015). Analysis of rice RNA-dependent RNA polymerase 6 (OsRDR6) gene in response to viral, bacterial and fungal pathogens. J. Gen. Plant Pathol..

[96] Canto-Pastor A., Santos B.A.M.C., Valli A.A., Summers W., Schornack S., Baulcombe D.C. (2019). Enhanced resistance to bacterial and oomycete pathogens by short tandem target mimic RNAs in tomato. Proc. Natl. Acad. Sci. USA.

[97] Boccara M., Sarazin A., Thiébeauld O., Jay F., Voinnet O., Navarro L., Colot V. (2014). The Arabidopsis miR472-RDR6 Silencing Pathway Modulates PAMP- and Effector-Triggered Immunity through the Post-transcriptional Control of Disease Resistance Genes. PLoS Pathog..

[98] Cai Q., Liang C., Wang S., Hou Y., Gao L., Liu L., He W., Ma W., Mo B., Chen X. (2018). The disease resistance protein SNC1 represses the biogenesis of microRNAs and phased siRNAs. Nat. Commun..

[99] Kong X., Yang M., Le B.H., He W., Hou Y. (2022). The master role of siRNAs in plant immunity. Mol. Plant Pathol..

[100] Ding S.-W., Voinnet O. (2007). Antiviral Immunity Directed by Small RNAs. Cell.

[101] Weiberg A., Jin H. (2015). Small RNAs—The secret agents in the plant–pathogen interactions. Curr. Opin. Plant Biol..

[102] Hamilton A.J., Baulcombe D.C. (1999). A Species of Small Antisense RNA in Posttranscriptional Gene Silencing in Plants. Science.

[103] Septiani P., Pramesti Y., Ghildan M., Aprilia K.Z., Awaludin R., Medina S., Subandiyah S., Meitha K. (2025). RNAi-based biocontrol for crops: A revised expectation for a non-recent technology. Planta.

[104] Hernández-Soto A., Chacón-Cerdas R. (2021). RNAi crop protection advances. Int. J. Mol. Sci..

[105] Bakhat N., Jiménez-Sánchez A., Ruiz-Jiménez L., Padilla-Roji I., Velasco L., Pérez-García A., Fernández-Ortuño D. (2025). Fungal effector genes involved in the suppression of chitin signaling as novel targets for the control of powdery mildew disease via a nontransgenic RNA interference approach. Pest Manag. Sci..

[106] Guo Z., Li Y., Ding S.-W. (2018). Small RNA-based antimicrobial immunity. Nat. Rev. Immunol..

[107] Brodersen P., Voinnet O. (2006). The diversity of RNA silencing pathways in plants. Trend Genet..

[108] Garcia-Ruiz H., Takeda A., Chapman E.J., Sullivan C.M., Fahlgren N., Brempelis K.J., Carrington J.C. (2010). *Arabidopsis* RNA-Dependent RNA Polymerases and Dicer-Like Proteins in Antiviral Defense and Small Interfering RNA Biogenesis during *Turnip Mosaic Virus* Infection. Plant Cell.

[109] Wang X.-B., Wu Q., Ito T., Cillo F., Li W.-X., Chen X., Yu J.-L., Ding S.-W. (2009). RNAi-mediated viral immunity requires amplification of virus-derived siRNAs in *Arabidopsis thaliana*. Proc. Natl. Acad. Sci. USA.

[110] Yang J., Zhang T., Li J., Wu N., Wu G., Chen X., He L., Chen J. (2019). *Chinese wheat mosaic virus*-derived vsiRNA-20 can regulate virus infection in wheat through inhibition of vacuolar- (H^+^)-PPase induced cell death. New Phytol..

[111] Liu P., Zhang X., Zhang F., Xu M., Ye Z., Wang K., Liu S., Han X., Cheng Y., Zhong K. (2021). A virus-derived siRNA activates plant immunity by interfering with ROS scavenging. Mol. Plant.

[112] Cao M., Du P., Wang X., Yu Y.-Q., Qiu Y.-H., Li W., Gal-On A., Zhou C., Li Y., Ding S.-W. (2014). Virus infection triggers widespread silencing of host genes by a distinct class of endogenous siRNAs in *Arabidopsis*. Proc. Natl. Acad. Sci. USA.

[113] Wang M., Dean R.A. (2020). Movement of small RNAs in and between plants and fungi. Mol. Plant Pathol..

[114] Li F., Pignatta D., Bendix C., Brunkard J.O., Cohn M.M., Tung J., Sun H., Kumar P., Baker B. (2012). MicroRNA regulation of plant innate immune receptors. Proc. Natl. Acad. Sci. USA.

[115] Lopez-Gomollon S. (2011). Detecting sRNAs by Northern blotting. Methods Mol. Biol..

[116] Koeppe S., Kawchuk L., Kalischuk M. (2023). RNA Interference Past and Future Applications in Plants. Int. J. Mol. Sci..

[117] Barrera-Figueroa B.E., Gao L., Wu Z., Zhou X., Zhu J., Jin H., Liu R., Zhu J.-K. (2012). High throughput sequencing reveals novel and abiotic stress-regulated microRNAs in the inflorescences of rice. BMC Plant Biol..

[118] Shivaprasad P.V., Chen H.-M., Patel K., Bond D.M., Santos B.A., Baulcombe D.C. (2012). A MicroRNA Superfamily Regulates Nucleotide Binding Site–Leucine-Rich Repeats and Other mRNAs. Plant Cell.

[119] Morgado L., Johannes F. (2017). Computational tools for plant small RNA detection and categorization. Briefings Bioinform..

[120] Thiebaut F., A Rojas C., Grativol C., Motta M.R., Vieira T., Regulski M., Martienssen R.A., Farinelli L., Hemerly A.S., Ferreira P.C. (2014). Genome-wide identification of microRNA and siRNA responsive to endophytic beneficial diazotrophic bacteria in maize. BMC Genom..

[121] Medina C., da Rocha M., Magliano M., Raptopoulo A., Marteu N., Lebrigand K., Abad P., Favery B., Jaubert-Possamai S. (2018). Characterization of siRNAs clusters in Arabidopsis thaliana galls induced by the root-knot nematode Meloidogyne incognita. BMC Genom..

[122] Ge F., Huang X., Hu H., Zhang Y., Li Z., Zou C., Peng H., Li L., Gao S., Pan G. (2017). Endogenous small interfering RNAs associated with maize embryonic callus formation. PLoS ONE.

[123] Ahmed W., Xia Y., Li R., Zhang H., Siddique K.H., Guo P. (2021). Identification and Analysis of Small Interfering RNAs Associated With Heat Stress in Flowering Chinese Cabbage Using High-Throughput Sequencing. Front. Genet..

[124] Yu D., Meng Y., Zuo Z., Xue J., Wang H. (2016). NATpipe: An integrative pipeline for systematical discovery of natural antisense transcripts (NATs) and phase-distributed nat-siRNAs from de novo assembled transcriptomes. Sci. Rep..

[125] Lei P., Qi N., Yan J., Zhu X., Liu X., Xuan Y., Fan H., Chen L., Duan Y., Wang Y. (2022). Genome-wide identification of small interfering RNAs from sRNA libraries constructed from soybean cyst nematode resistant and susceptible cultivars. Gene.

[126] Guo Q., Qu X., Jin W. (2014). PhaseTank: Genome-wide computational identification of phasiRNAs and their regulatory cascades. Bioinformatics.

[127] Fu K., Wu Q., Jiang N., Hu S., Ye H., Hu Y., Li L., Li T., Sun Z. (2023). Identification and Expressional Analysis of siRNAs Responsive to *Fusarium graminearum* Infection in Wheat. Int. J. Mol. Sci..

[128] Xu W.-B., Cao F., Liu P., Yan K., Guo Q.-H. (2024). The multifaceted role of RNA-based regulation in plant stress memory. Front. Plant Sci..

[129] Mierziak J., Wojtasik W. (2024). Epigenetic weapons of plants against fungal pathogens. BMC Plant Biol..

[130] Jain H., Kaur R., Sain S.K., Siwach P. (2024). Development, Design, and Application of Efficient siRNAs Against Cotton Leaf Curl Virus-Betasatellite Complex to Mediate Resistance Against Cotton Leaf Curl Disease. Indian J. Microbiol..

[131] Ali A., Shahbaz M., Ölmez F., Fatima N., Umar U.U.D., Ali A., Akram M., Seelan J.S.S., Baloch F.S. (2024). RNA interference: A promising biotechnological approach to combat plant pathogens, mechanism and future prospects. World J. Microbiol. Biotechnol..

[132] Deng P., Muhammad S., Cao M., Wu L. (2018). Biogenesis and regulatory hierarchy of phased small interfering RNAs in plants. Plant Biotechnol. J..

[133] Curaba J., Singh M.B., Bhalla P.L. (2014). miRNAs in the crosstalk between phytohormone signalling pathways. J. Exp. Bot..

